# Alzheimer Caregiving Problems According to ADLs: Evidence from Facebook Support Groups

**DOI:** 10.3390/ijerph19116423

**Published:** 2022-05-25

**Authors:** Pavel Bachmann, Jan Hruska

**Affiliations:** 1 Department of Management, University of Hradec Králové, 500 03 Hradec Králové, Czech Republic; 2 Department of Economy, University of Hradec Králové, 500 03 Hradec Králové, Czech Republic; jan.hruska.3@uhk.cz

**Keywords:** informal caregiving, Alzheimer’s disease, caregiver burden, activities of daily living, Facebook

## Abstract

*Background and study goal:* Social media are a societal phenomenon today, including the oldest generation, yet they are seldom used in current health research to identify the needs of persons with Alzheimer’s disease (PADs) and their carers. There is an even bigger research gap in the analysis of caregivers’ communication in online support groups and its classification according to activities of daily living (ADLs). For this, the goal of this study is to identify real-life practices of informal caregivers who care for PADs based on the analysis of their communication in Facebook groups. *Methods:* A sample of 1603 contributions was obtained from support groups by keyword search, manual coding, and verification; thus, the contributions in the sample are relevant for the individual basic ADLs of PADs. Next, five main conversation topics were identified for each ADL. This was done using the topic extractor based on simple parallel threaded implementation of LDA with a sparse sampling scheme and data structure. *Results:* The qualitative dimension of research identified discussion topics as well as specific caregiver behavior patterns for each ADL. The quantitative dimension determining the level of engagement of group members in individual ADLs was also measured. The highest engagement was found in activities of feeding and drinking, followed by bathing. In contrast, the activities of dressing, continence, and toileting attract the lowest interest. Moreover, the causal links between the topics discussed within the areas of ADLs were identified. *Conclusions:* The acquired knowledge can help further research focus on the most problematic areas relevant for people with AD in order to increase their quality of life and at the same time reduce the caregiver burden. The study expands the information of the demands posed by the individual caregiver activities, specifically in the context of activity-based costing or time-based activity costing.

## 1. Introduction

Alzheimer’s disease (AD) is one of the greatest global health challenges of this century, as over 47 million people are estimated to be living with AD or related dementia [1]. Weller and Budson [2] characterize AD as a neurodegenerative process usually characterized by β-amyloid plaque deposition and neurofibrillary tangles of hyperphosphorylated tau. Diagnosis is based upon clinical presentation fulfilling several criteria as well as fluid and imaging biomarkers. Treatment is currently targeted toward symptomatic therapy, although trials are underway that aim to reduce the production and overall burden of pathology within the brain. A clinical condition is characterized by an acquired decline in mental ability that eventually becomes severe enough to increase the person’s dependence on external care, especially as such provided in the family environment. Beyond exhibiting cognitive impairments, persons with AD (PADs) frequently present behavioral symptoms such as depression, irritability, aggression, sleep disturbances, and so on [3].

The above-mentioned clinical and behavioral manifestations of the disease have a significant impact not only on PADs but also on their immediate surroundings, especially primary informal caregivers and their burden. In AD, the main aim of the treatment might be to enhance the quality of life (QoL) by delaying the development of the disease and maintaining the person’s ability in activities of daily living (ADLs). The concept of ADLs was proposed in 1950 by Sidney Katz and his team to measure a person’s ability or inability to perform individual activities [4,5]. Basic ADLs currently include bathing and showering, personal hygiene, dressing, toilet hygiene, functional mobility, and self-feeding [6]. This basic ADLs concept was early extended by instrumental frameworks [7] or later by occupational frameworks [8] and others. At the same time, the current form of informal care for the oldest generation is changing in several respects. In terms of the work of caregivers, there is an obvious transition to online communication, especially through social networks. The already-mentioned transition to the online environment has recently been exacerbated by the current pandemic, which has caused a mass shift to the online experience, including populations of older adults.

Despite the fact the social media are a societal phenomenon today, the research gap exists in the use of online support groups for evidence to better characterize caregivers’ problems specifically according to individual ADLs. Therefore, the present study aims to identify the content of communication of informal caregivers in relation to the individual basic ADLs.

### 1.1. Caregiver Burden

Caregiver burden has been defined as a “stress response elicited by negative self-appraisal of coping capacity and/or resources needed to meet demands of caregiving” [9] or as an outcome of the interaction between characteristics of the PAD and the caregiver, where both of them can act as determinants [10]. Informal caregivers can be socially isolated and burdened during their long-term care for their loved ones [11].

Research studies particularly deal with the caregiver burden experienced by informal caregivers looking after PADs [11,12,13]. A meta-analysis has found that the caregivers of PADs are significantly more stressed than caregivers for persons without neurodegenerative diseases. These caregivers also suffer from more serious depressive symptoms and physical problems [14], specifically, the overall prevalence rate of depressive and anxiety symptoms is 34% and 44%, respectively [15]. The physical and psychological burden of providing assistance with ADLs can severely influence not only the informal carers but also the whole family [16,17]. Caregivers are forced to limit the time for their own self-care and socialization [18] and are exposed to a higher risk of depression and health problems in general [19,20]. Bachmann [21] identified the framework of caregivers’ daily issues including recognition or non-recognition of close family relatives, exhaustion and feelings of resignation, fear of the future, and other caregiver issues.

Despite the fact that PADs in the late stages are not often involved in research studies, the burden grows with the progression of the disease due to the loss of basic ADLs and the person’s specific behavior, as agitation, aggression, and disinhibition, followed by delusions and mood disturbances [22,23].

### 1.2. Use of Social Media for Healthcare Information Search

The benefits of social media (SM) use as reported by adults seeking healthcare information are interactions with others with the same condition, the increased availability of information, and emotional support [24]. In a survey of caregivers’ online health behaviors [25], caregivers used the internet to obtain health information more than non-caregivers, at 72% and 50%, respectively; furthermore, 52% of caregivers participated in online social activity as compared with 33% of non-caregivers.

### 1.3. Social Media Caregiver Support

Primarily, the caregivers feel empowered by the support group experience that allows for intimacy and bonding [25,26]. Moreover, SM also provide new ways to assist PADs, nurses, and families in gaining support in various life and health situations [27]. Informal caregivers use SM to share their daily practice. At the same time, by understanding the ways of SM use as a part of the caregiving experience, interventions and services aimed at improving caregiver burden and quality of life can be developed [28]. It is also worth noting the dangers of social media use in healthcare research, specified by Ventola [29] as a reduced quality of information, the posting of unprofessional content, forming the first impression based on the user’s personality and not on the content, breaching people’s privacy, violating the boundary between patients and healthcare professionals, licensing, and legal issues.

### 1.4. Effectiveness of Social Media Support

The use and effectiveness of social media in supporting persons with disease and their caregivers are mentioned relatively frequently in the literature [14,15]. With more common diseases, such as cancer or arthritis, the discussion support groups are used by the persons with disease themselves [30]. However, with older adults’ diseases, the discussion groups serve rather as a support for the caregivers and family members [30,31]. While in general, the health content on social media is rather impersonal and related to marketing [32], the private discussions on Facebook are more suitable for delivering support to the caregivers of PADs [24,26].

## 2. Methods

### 2.1. Research Questions

The Facebook support groups provide a very large database of daily situations, emotions, and experiences that describe the relationships between the caregivers and PADs, which we—for the purpose of this study—call “caregiver problem”. For this reason, it might be very fruitful to interconnect such evidence on caregiver problems with the ADLs classification. Therefore, the research questions are formulated as:

RQ1: What are the caregiver problems faced by caregivers of PADs within ADLs?

RQ2: Are there causal links between caregiver problems within or across ADLs?

### 2.2. Sample

The research sample was obtained from the communication of caregivers in two Facebook groups, “Molly’s Movement” and “Alzheimer’s and Dementia Support”. These specific groups were selected with respect to the representativeness of the sample. In terms of membership, these are the largest support groups worldwide, focused mainly on AD (the overall group topics included the tags +alzheimers, +alzheimerawareness, +dementia, and +dementiacare), are open to all countries and therefore have an international character, the language used is English, so there is no distortion of communication content. Last but not least, these groups show high activity in the number of published posts and interactions on them in the form of comments and reactions.

The selection of posts for the research sample was made depending on the keywords representing the individual basic ADLs. Posts that were not directly related to either AD or considered daily activity were not selected. The search took place through a search engine, which is incorporated as a part of Facebook groups. The total amount of 1603 posts published by caregivers between July 2017 and December 2019 were collected, processed, and coded both manually and with the help of machine learning. Selected descriptive statistics of obtained data are available in Table 1.

The caregiver problems in the posts sampled documented either multiple problems or one specific area. In the first type of post, the caregivers often provided a detailed description of their relationship with the PAD, their health condition and complications, and their age or length of care. More specifically focused posts concerned specific problems, experience, feelings, or questions.

### 2.3. Data Collection

The data were obtained by entering keywords representing the basic ADLs in a search box. When a post was found, then read to verify that it is logically correct and pertinent to the specific ADL. Verification was necessary due to the ambiguity of some words (e.g., moving, dressing). After this verification the data were downloaded to MS Excel processing editor. The data obtained included the text of the post, the date of its publication, the number of reactions, and the number of comments. A summary of the keywords used and the relevant caregivers’ posts is available in Table 2.

### 2.4. Data Processing

A qualitative analysis was based on the inductive approach and elements of the grounded-theory approach (codes, concepts, categories, theory).

**Figure 1:**
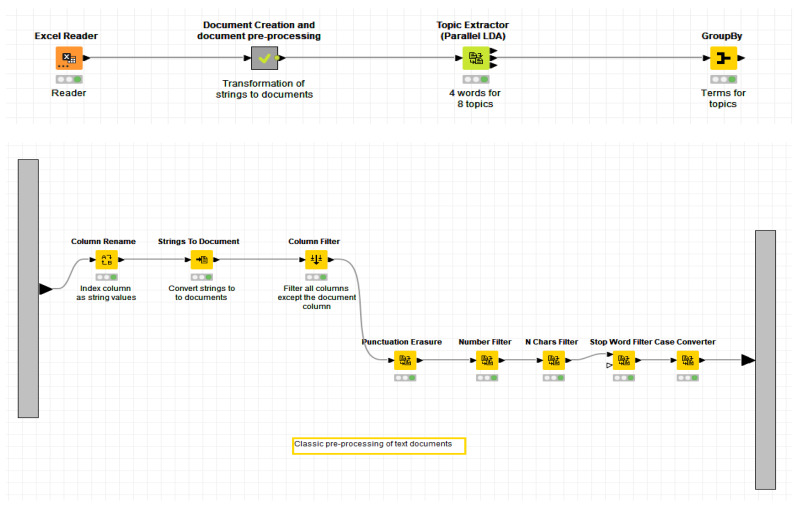
A description of the data editing workflow in the KNIME software to identify the main areas discussed (Source: KNIME Analytics Platform, Topic Detection Analysis).

First, the individual posts were read and manually coded and categorized under the relevant groups of ADLs. Second, the conversation topics were identified separately for each ADLs. For this purpose, the analytical software KNIME was used, which integrates various components for machine learning and data mining. KNIME as an analytical platform was designed to gather, visualize, manage, and optimize data. Main conversation topics were identified using the topic extractor based on simple parallel threaded implementation of latent Dirichlet allocation (LDA) following Newman, Asuncion, Smyth, and Welling [33] with sparse LDA sampling scheme and data structure from Yao, Mimno, and McCallum [34]. The nodes used in the workflow use a topic modeling library based on machine learning. A description of the workflow is available in Figure 1.

**Figure 2:**
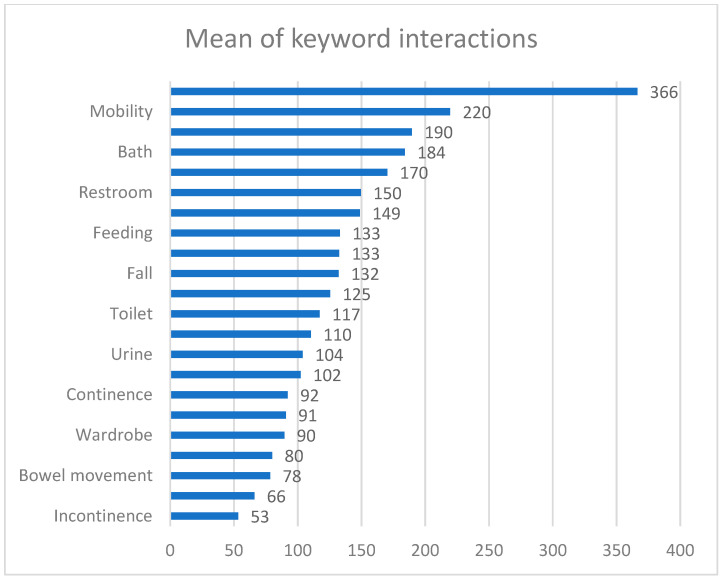
Overview of keywords and their interactions.

Third, an engagement of the group members in individual ADLs with descriptive statistics was calculated (see Table 9). Fourth, an analysis of variance (ANOVA) was used to identify significant differences in the frequency of communication about ADLs as well as among the most common keywords (see Table 10 and Figure 2). Fifth, the topics detected through machine learning were re-examined and re-verified by both authors. The most common behavior or communication patterns representing caregiver problems were assigned to each discussion topic detected (see Tables 3–8). Sixth, a detailed analysis of the identified typical problems and situations enabled to find basic recurring causal links (Table 11).

### 2.5. Ethical Aspects of Data Collection and Processing

Both groups have their content set to public, which means that anyone can find the group. When sending a request to join the group, the researchers disclosed to the group administrators our interest in conducting research in the field of caregivers and their behavior on social networks. The data processing was strictly anonymous, and no personal data of any of the group members were used in the research.

## 3. Results

### 3.1. Qualitative Dimension

#### 3.1.1. Discussion Topics for the “Bathing” Activity

Caregivers seek help from other users regarding hygiene, which can be generally lower. PADs sometimes refuse to bathe, forget, and need assistance during bathing. Along with hygiene, the caregivers also communicate various infectious diseases among their concerns. The identified discussion topics and relevant examples posted by the caregivers are available in Table 3.

#### 3.1.2. Discussion Topics for the “Dressing” Activity

Discussion topics in the field of dressing were relatively diverse and focused on comfortable dressing, the ability of PADs to choose clothes appropriate to the situation (day and night, formal wear) or feelings of cold.

The identified discussion topics and relevant examples posted by the caregivers are available in Table 4.

#### 3.1.3. Discussion Topics for the “Toileting” Activity

The topic of toileting is similar in its nature to bathing. The discussion topics include, for example, repeated visits to the toilet and the consequent frustration of the caregiver resulting from the time and mental demands, as well as physical demands, where assistance with using the toilet is required. At the same time, caregivers often mention the refusal of PADs to go to the toilet and the related verbal conflicts, the inability to use the toilet, or the inability to perform a UTI test.

The identified discussion topics and relevant examples posted by the caregivers are available in Table 5.

#### 3.1.4. Discussion Topics for the “Transferring/Mobility” Activity

Discussion topics identified for the transferring/mobility activity often included areas with difficult access (stairs), limitation of the PAD’s independence, and falls and subsequent pain and health complications.

The identified discussion topics and relevant examples posted by the caregivers are available in Table 6.

#### 3.1.5. Discussion Topics for the “Continence” Activity

In the area of continence, the most discussed topic was the need to use continence wear and the need for surgical solutions to incontinence problems. Given the deteriorating condition in this area, caregivers often asked about the right time or situation to place the PAD in a medical facility or a hospice. The identified discussion topics and relevant examples posted by the caregivers are available in Table 7.

#### 3.1.6. Discussion Topics for the “Feeding and Drinking” Activity

Caregivers mention weight loss in PADs due to insufficient eating and drinking and difficulties eating, such as dry mouth or pain when swallowing. Furthermore, PADs often completely refuse food and drink, and have trouble sleeping. Along with poor eating patterns, there are other health problems as well, and caregivers ask how others tackle the problem. The identified discussion topics and relevant examples posted by the caregivers are available in Table 8.

### 3.2. Quantitative Dimension

#### Engagement of Group Members with Content Related to the Individual ADLs

In general, social media engagement rate is expressed as a combination of reactions (labeled on Facebook as likes, haha, sad, super, and wow reactions), comments, and shares. Facebook groups usually limit the sharing function, as it is impossible to share a group post with non-members. From the point of view of the members’ interest, writing a comment is a manifestation of a higher interest rather than simply clicking a reaction. It is more time-consuming and mentally more demanding to write such a comment, and at the same time, the comment can usually provide more support for the author of the post. Therefore, for the purposes of our study, the weight of a comment is double the weight of a reaction.

In the examined posts, it was found that the members of the group generated an average of 60 (59.73) reactions and 40 (39.75) comments per post. This is a significantly lower number of reactions and a higher number of comments than what is generally the case in other Facebook groups. It seems that the difference between the number of reactions and comments is likely to be affected by the intimacy of the topic, as the lowest differences were recorded for ADLs related to toileting and continence.

The highest total engagement of group members was recorded with activities related to feeding and drinking (217.41). On the contrary, the lowest interest of the members was found in activities related to dressing (111.46) and continence (117.77). Statistically significant differences in member engagements were found in the area of drinking and feeding. Statistical analysis was performed using ANOVA, while the conservative Bonferroni post-hoc test was used to determine differences. In the calculation of the engagement rate, the weight assigned to the comments was increased as it is a common practice. The “like” reaction represents only a single click, while making a comment requires much higher involvement of the user (members try to advise or encourage and they usually write a few sentences [35]). The results of testing are available in Table 9.

A statistical analysis among the individual ADLs categories shows that the most significant differences exist in the engagement between feeding and continence (*p* = 0.006), feeding and dressing (*p* = 0.007), and feeding and toileting (*p* = 0.020). Analysis of variance (ANOVA) and the conservative Bonferroni post-hoc test in the SPSS software were used for the statistical analyses. The results of testing are available in Table 10.

The only statistical difference in the ANOVA test was between the word “drinking” and the words “bath, bowel, bowel movement, clothing, continence, diapers, dress, eating, fall, incontinence, move, restroom, shower, toilet, wardrobe and wear”.

Figure 2 shows the keyword “drinking” with the highest number of interactions. This is a basic human need, and when PADs stop drinking due to pain or forgetting, caregivers often fear for the lives of their loved ones and often share their frustration on social networks, where they seek support.

## 4. Discussion and Conclusions

### 4.1. Main Findings

The study documents the real practice of carers of PADs in two dimensions: qualitative and quantitative.

Qualitative research found that the main problems were associated with refusing to carry out daily regular activities (especially showering, washing, and dressing), reduced levels of hygiene (inappropriate use of toilets, incontinence, and keeping the house clean), and health concerns about the PAD (malnutrition, falling and especially night or stair falls). At the same time, the caregivers were concerned about financial costs increase (toilet repairs, increased material costs and frequent laundry). The discussion topics were identified for each of the ADLs, including real problems documenting PADs and the caregiver relationship and caregiver burden (for more detail see Table 3, Table 4, Table 5, Table 6, Table 7 and Table 8). The quantitative dimension research showed that out of the six activities analyzed, the activity of feeding and drinking, followed by bathing, draws the highest engagement of other members.

Moreover, the high number of qualitative findings enabled us to find causal connections between individual ADLs. The basic causalities revealed by the research are summarized in Table 11. Of course, there may exist more of these causalities than indicated, and the ones may overlap or be applicable to more ADLs.

A synthesis of the ADLs discussed also shows that some problems in the care for PADs are recurrent. These recurring problems include the refusal to perform some ADLs (most apparent in showering and going to the toilet) and the need for the caregiver to provide precise instructions or to supervise the activities. The caregivers communicated their frustration, tiredness, and uncertainty. In certain situations, they have to remodel their housing to make it safer and more suitable for the patient.

The study findings can be supported by a longitudinal study of Saari et al. [36], which found symptom reactions as apathy, appetite disturbances, and aberrant motor behavior in PADs. The similar results were provided earlier in a more general study of [37] on neurodegenerative diseases, which confirmed the high emotional burden of PADs caregivers. Furthermore, the study underlines and extends several earlier research findings. At first, the Facebook discussion groups can be considered as a suitable platform for making qualitatively oriented research [38] and they can have a positive effect on social support and the self-efficacy of caregivers [16]. At second, the research findings support Lagervall et al. [39] assertion that Facebook groups encourage mental well-being and are particularly advantageous for caregivers of individuals diagnosed with dementia, as it is difficult to leave these persons alone.

### 4.2. Limitations

The limitations of this study include an insufficient sample size for statistical measurement. Although for each ADL more than 200 posts were obtained, it is still possible that the caregiver problems would have turned out differently if more posts had been obtained. For this reason, the study findings can have only a limited generatability. Moreover, it is also possible that the topics discussed develop over time, so another limitation of the study may be the fact that our sample only covers the period of the last four years.

## 5. Practical and Theoretical Implications and Future Directions

At the practical level, the present study provides an insight into real-life everyday situations of informal caregivers, defining the main behavior patterns of PADs and at the same time identifying important needs and issues of the caregivers. For further research, the study may contribute to the knowledge for a further distinction of the complexity of the individual caregiving activities, specifically in the implementation of activity-based costing or time-based activity costing. At the same time, the study can help in identifying the needs of caregivers in the field of innovation development or the implementation of online counseling on social networks.

Methodologically, the study is original in that it brings a new type of data, which will probably play a much more significant role in the future. The method of data processing can be easily repeated, and the workflow used for the analysis of discussion topics is freely available. The information obtained in our study has the potential to identify the most problematic areas in PADs and thus enable the improvement of QoL of both PADs and their caregivers in the future, ultimately improving the overall socioeconomic situation of households and the society as a whole.

Following this study, the authors plan to conduct similar research focused on other widespread neurodegenerative diseases, including Parkinson’s disease, amyotrophic lateral sclerosis, and vascular dementia. We would strongly suggest calling other researchers to use online social support groups analysis to effectively reveal (and consequently address) the vital problems of caregivers of persons with neurodegenerative diseases.

## Figures and Tables

**Table 1:** Characteristics of the sample of caregivers and PADs.

*Caregiver’s gender*	69.9% woman; 30.1% man
*PAD’s gender*	64% woman; 34% man; 2% unknown
*PAD’s relationship status towards the caregiver*	50% mother; 14% father; 10% wife/husband; 8% mother-/father-in-law; 4% grandparents; 4% uncle or aunt; 10% unknown
*Caregiver’s country of residence*	95% United States; 1% United Kingdom; 1% Canada; 3% other countries or unknown

**Table 2:** Katz Index of Independence in ADL and its keywords (Source: https://www.cfps.org.sg/publications/the-singapore-family-physician/article/221_pdf, accessed on 5 February 2022).

ADLs	*N*	Search Keywords
*Bathing/personal hygiene*	376	bath, shower, clean
*Dressing*	208	dress, clothes, wardrobe, wear
*Toileting*	299	toilet, restroom, latrine
*Transferring/mobility*	234	transfer, shift, move, movement, transport, moving, mobility, motion, stairs
*Continence*	284	bowel movement, BM, incontinence, urine, continence, bowel, diapers
*Feeding*	202	eating, drinking, chewing, consuming, dining

**Table 3:** Overview of discussion topics for the “bathing” activity.

Discussion Topic	Automatically Detected Discussion Words *	Problems Mentioned in the Group
1: The need to wash/shower associated with the poor hygiene of PADs.	Mom, poo, help, told, towel, fight, months, don’t, anymore, remember, God, giving, nasty.	Bad hygiene/odor of the PAD. Frequency of bath/shower time. Relief that the PAD’s shower/bath was successfully finished.
2: Refusing to have a bath.	Time, mom, help, day, bed, eyebrows, chair, refuses, stage, sign, standing, pooping, recommendation.	How to persuade a person to wash/take a shower. Mentioning the time since the last shower and determining the frequency of washing in others. A person is mean or agitated when asked to go to the shower.
3: Repetitive bathroom visits.	Times, walk, soap, fall, husband, tonight, getting, water, bed, using, remember, mom, clothes, dementia, ready, day.	Bathing obsession. Falls related to frequent bathroom visits.
4: Assistance during taking a bath.	Toilet, tried, help, morning, dad, day, woke, shampoo, loved, urine, porch, continue, assistance.	Higher need for assistance. Searching for tips how to simplify the process; where to find an external help. Lack of physical preparedness of the caregiver. Physical inability of the PAD to go to the bathroom.
5: Insufficient cleanliness associated with advanced disease progression.	Smell, check, bad, diaper, hubby, bedroom, home, house, weeks, help, mom, clean, night, help, dad, hallucinating.	Bad odor and low overall hygiene of the house. Low hygiene/cleanliness in specific places in the house.

* Words directly related to the activity (bath, bathe, bathed, shower, and bathroom) were excluded from the table.

**Table 4:** Overview of discussion topics for the “dressing” activity.

Discussion Topic	Automatically Detected Discussion Words *	Problems Mentioned in the Group
1: Suitable clothing.	Day, home, morning, night, people, can’t, fell, lives, grandad, nan, toilet, bath, depends, catheter, hospital.	Search for more comfortable clothes (without seams). Bad dressing choices. Anti-strip jumpsuits—recommendations. How to dress a person with a catheter or other health disadvantages. Constant undressing (including unsuitable/public places and situations).
2: Burden associated with frequent laundry (including bed linen).	Ago, laundry, couple, towels, washing, daily, enjoyed, getting, stubborn, eating, close, independent, feel.	Time demands and financial costs associated with laundry (bed sheets, underwear, socks). Losing clothes.
3: Dressing for special occasions.	Shower, day, mom, time, pearls, putting, else, dad, house, care, eat, various, finally, life.	Dressing for eating out, anniversaries. Using tracking wristbands as an accessory when leaving the house.
4: Feeling cold.	Cold, reason, home, dementia, mom, stern, ready, trying, told, tried, head, underpants, stage, normal.	PADs’ demands for warm clothes. Caregivers’ concerns about PADs overheating.
5: Refusal to change one’s clothes.	Bed, change, pajamas, told, turtleneck, laying, jeans, otherwise, time, depends, gone, bring, day, help, morning.	Staying the whole day in pajamas Refusing to wear nightwear. Refusing to dress.

* Words directly related to the activity (dressed, dresses, clothes, wear, and wearing) were excluded from the table.

**Table 5:** Overview of discussion topics for the “toileting” activity.

Discussion Topic	Automatically Detected Discussion Words	Problems Mentioned in the Group
1: Toilet paper hoarding/stealing.	Paper, rolls, pants, found, pee, day, told, lol, wipe, started, time, putting, peed, depends, attempting, random, upset, husband.	Regular hoarding/hiding of toilet paper by PADs. Concerns of PADs that someone is stealing toilet paper. “Thefts” of toilet paper in a hospital facility.
2: Issues with hygiene after using the toilet.	Help, father, trying, tried, issue, pants, understand, floor, seat, stand, pooping, night, concerned, pee.	Refusal to wipe after bowel movement; dirty toilet paper on the backside. Disposing of used toilet paper outside the toilet (bathroom bin). How to help PADs with wiping.
3: Improper use of the toilet.	Roll, flushing, tissue, dementia, night, tonight, water, carpet, pull, hands, ago, day, times, weeks, care, bedroom.	Clogging the toilet bowl with toilet paper. Throwing various objects, clothes (underwear), and food into the toilet bowl. Repeated flushing. Fears of flooding; associated repair costs (plumber).
4: Urinating/defecating outside of the toilet/bathroom.	Clean, else, help, advice, try, time, doing, stop, house, thank, ideas, trash, wipes, mother, helping, urinates, getting, dad.	Most often the bathroom bin or the bathroom floor. Various places in the house. Subsequent tracking of urine/stool around the house.
5: Inability or refusal to go to the toilet.	Day, time, underwear, unit, poor, trying, feces, week, month, difficult, attention, soon, throughout, recently, UTI, test.	Consequences for caregivers, caregivers’ health concerns. Health consequences for PADs. Conflicts (verbal, physical) related to the problem.

**Table 6:** Overview of discussion topics for the “transferring/mobility” activity.

Discussion Topic	Automatically Detected Discussion Words *	Problems Mentioned In The Group
1: Poor mobility on stairs.	Hand, morning, night, life, downstairs, time, husband, door, can’t, help, stairs, months, fast, force, keeping, angry, hold.	Falling on stairs due to bad balance. Waking up at night and walking around the house. Hallucinations and feelings of anger. PADs being angry with family members because they give them orders what to do.
2: Decline in mobility, falling more often, refusing to use a walker.	Care, falling, spinal, dementia, doctors, stop, days, hospice, slow, pain, yesterday, causing, cane, walker, stroke, feet, front, hold, unable.	Falling from a wheelchair due to loss of mobility/stroke/feeling weak. Not being able to walk, hesitating to walk.
3: Inability to move alone, need for hospital care, need for a wheelchair.	Home, nursing, dementia, fall, mobility, hospital, able, help, stage, looking, care, disease, days, eating, wheelchair, gone, suggestions, sleep, told.	Not being able to move to or from a wheelchair. Discussing other diseases connected with falling/not walking. Talking about when it is the right time to move the PAD to a hospital/hospice.
4: Bathroom falls and night falls (stairs).	Weeks, Alzheimer, started, bathroom, night, help, falls, thank, week, hospital, please, dementia, found, days, brother, stages, call.	Feeling dizzy, paranoia, anxiety in everyday routine. Memory issues with basic tasks.
5: Hurting legs after bad mobility/fall.	Dementia, time, walk, care, memory, falling, home, issues, feel, Alzheimer’s, legs, facility, don’t, term, living, mother, diagnosed, won’t.	Desperate about the situation (don not know what to do). Falling more often because of more severe stages.

* Words directly related to the activity (move, moved, movement, move, and mobility) were excluded from the table.

**Table 7:** Overview of discussion topics for the “continence” activity.

Discussion Topic	Automatically Detected Discussion Words *	Problems Mentioned in the Group
1: Need to use continence wear.	Diapers, adult, time, suggestions, depends, help, night, issues, thank, stage, wear, overnight, thanks, looking, advice, skin, briefs, reason.	PADs get up at night and wander around the house. PADs refuse to use continence wear. Caregivers feeling desperate about “poop disaster”.
2: Need surgery for incontinence problems.	Home, hospital, care, help, dementia, bowel, she’s, he’s, started, surgery, days, feel, lost, nursing, loved, stage, husband, doctor, family, ones.	Staying in hospital after surgery. Infection/fever/lungs/blocked bowel problems.
3: Added more health problems after incontinence issues, need to be placed in a facility.	Dementia, care, sleep, memory, issues, person, time, people, help, change, facility, getting, caregiver, yesterday, times, diarrhea, health, dose	Unable to sleep. Don not recognize family members. Asking the group when it is time to move the PAD to a medical facility.
4: Incontinence problems associated. with eating and drinking.	Bowel, days, time, hospice, movement, week, eating, home, mother, nurse, food, months, control, little, drink, she’s, morning, sometimes, weeks.	Forget to drink and eat/do not want to eat because of a sore throat. Urinating everywhere in the house.
5: Constant urine problems, need to use continence wear, UTI.	Bowel, time, diapers, help, toilet, taking, clothes, getting, movement, morning, shower, else, floor, clean, advice, care, constantly, urine.	Asking the group what continence wear are the most suitable. Continence problems in shower.

* Words directly related to the activity (incontinence and continence) were excluded from the table.

**Table 8:** Overview of discussion topics for the “feeding” activity.

Discussion Topic	Automatically Detected Discussion Words *	Problems Mentioned in the Group
1: Losing weight.	Week, barely, stages, times, days, pounds, tube, meals, half, grandma, swallow, she’s, love, lost, dementia, trying.	Want to sleep instead of eating. Eat only one bite or take just a sip of water.
2: Problems with swallowing.	Home, getting, nursing, stage, hospital, trying, Friday, stove, plate, meds, question, months, cancer, swallowing, sister, people.	Unable to chew or swallow because of pain. Caregivers share how the PAD passed away (share symptoms). PADs experience dry/sore throat.
3: Stopped eating and drinking, sleep problems.	Time, hospice, care, dementia, weeks, days, doctor, night, yesterday, stopped, hospital, sleep, little, mother, past, disease, home.	Caregivers worry about the PAD dying because they refuse to eat and drink. PADs stay in bed for weeks.
4: Forget about eating or drinking.	Water, sometimes, clothes, time, dinner, dementia, tell, coke, brain, lungs, mouth, break, hard, advice, tried, stages.	Do not remember their last meal/drink. Caregivers asking for advice how to make the PAD eat/drink.
5: Having other health problems more or less connected with poor eating and drinking patterns.	Days, home, bathroom, time, she’s, night, able, started, weeks, cough, nurse, doing, little, maybe, hours, covid, blood.	Caregivers feeling desperate and frustrated about the PAD. Asking group when to move the PAD to a hospital or hospice.

* Words directly related to the activity (eating, feeding, drinking, food, and dining) were excluded from the table.

**Table 9:** Engagement of group members with content related to the individual ADLs.

ADLs Category	N	Likes per Post (Mean)	Comments per Post (Mean)	Overall Engagement (Mean)	Std. Deviation	Std. Error	95% Confidence Interval for Mean	
*Bathing*	376	129.00	51.19	185.55	345.33185	17.80913	150.5349	220.5715
*Dressing*	208	58.07	32.38	111.46	234.19299	16.23836	79.4430	143.4705
*Toileting*	299	47.49	40.11	127.67	148.30209	8.57653	110.7940	144.5505
*Transferring*	234	133.85	34.24	133.29	359.78496	23.51988	86.9475	179.6251
*Continence*	284	49.08	38.63	117.77	176.98192	10.50195	97.0993	138.4430
*Feeding*	202	308.37	49.05	217.4158	492.07714	34.62243	149.1461	285.6856
In total	1603	59.7	39.7	149.5190	308.56894	7.70700	134.4022	164.6359

Descriptions of categories for likes and comments (weight of comments on “overall engagement mean” is multiplied by two).

**Table 10:** Differences between groups with ANOVA and Bonferroni post-hoc test.

	*Bathing*	*Dressing*	*Toileting*	*Transferring*	*Continence*	*Feeding*
***Bathing***	X	0.079	0.225	0.614	0.075	1
***Dressing***	0.079	X	1	1	1	0.007 *
***Toileting***	0.225	1	X	1	1	0.020 *
***Transferring***	0.614	1	1	X	1	0.065
***Continence***	0.075	1	1	1	X	0.006 *
***Feeding***	1	0.007 *	0.020 *	0.065	0.006 *	X

* Significant differences of engagement in communication about specific ADLs.

**Table 11:** Basic causalities according to ADLs.

*Bathing*	advanced disease progression + rejection of bath/shower → need of assistance during taking a bath + and/or insufficient home cleanliness → lower personal hygiene of PAD
*Dressing*	feeling cold + refusal to change clothes → lower personal hygiene of PAD
*Toileting*	improper use of toilet (repetitive flushing, throwing objects or clothes into the toilet) + toilet paper hoarding/stealing → financial concerns low personal hygiene after the use of toilet + urination and defecation outside the toilet → lower personal hygiene of PAD → insufficient home cleanliness
*Transferring*	refusing to use a walker + and/or stairs/bathroom/night falls → not able to move alone + hurting legs after mobility/fall → not able to move alone → need of a wheelchair + and/or need of hospital care
*Continence*	constant urine problems + problems associated with feeding and drinking → incontinence problems → need to use continence wear + and/or need of hospital care + UTI treatment or surgery
*Feeding and drinking*	problems with swallowing + and too much sleeping + low nutrition of received food → loss of weight → concerns of caregivers on proper feeding and drinking

## Data Availability

Not applicable.
